# Use of flaps in inguinal lymphadenectomy in metastatic penile cancer

**DOI:** 10.1590/S1677-5538.IBJU.2021.99.14

**Published:** 2021-06-10

**Authors:** Roberta Alvares Azevedo, Ana Claudia Roxo, Silvia Helena Baima Alvares, Daniel Pereira Baptista, Luciano A. Favorito

**Affiliations:** 1 Hospital Mário Kröeff Divisão de Cirurgia Plástica Rio de JaneiroRJ Brasil Divisão de Cirurgia Plástica, Hospital Mário Kröeff, Rio de Janeiro, RJ, Brasil; 2 Universidade do Estado do Rio de Janeiro Departamento de Cirurgia Geral Rio de JaneiroRJ Brasil Departamento de Cirurgia Geral, Universidade do Estado do Rio de Janeiro – UERJ, Rio de Janeiro, RJ, Brasil; 3 Universidade do Estado do Rio de Janeiro Divisão de Cirurgia Plástica Rio de JaneiroRJ Brasil Divisão de Cirurgia Plástica, Departamento de Cirurgia Geral, Universidade do Estado do Rio de Janeiro – UERJ, Rio de Janeiro, RJ, Brasil; 4 Universidade do Estado do Rio de Janeiro Unidade de Pesquisa Urogenital Rio de JaneiroRJ Brasil Unidade de Pesquisa Urogenital, Universidade do Estado do Rio de Janeiro – UERJ, Rio de Janeiro, RJ, Brasil

**Keywords:** Penile Neoplasms, Lymph Node Excision, Lymphatic Metastasis

## Abstract

**Purpose:**

Reviewing surgical procedures using fasciocutaneous and myocutaneous flaps for inguinal reconstruction after lymphadenectomy in metastatic penile cancer.

**Material and Methods:**

We reviewed the current literature of the Pubmed database according to PRISMA guidelines. The search terms used were “advanced penile cancer”, “groin reconstruction”, and “inguinal reconstruction”, both alone and in combination. The bibliographic references used in the selected articles were also analyzed to include recent articles into our research.

**Results:**

A total of 54 studies were included in this review. About one third of penile cancers are diagnosed with locally advanced disease, often presenting with large lymph node involvement. Defects in the inguinal region resulting from the treatment of metastatic penile cancer are challenging for the surgeon and cause high patient morbidity, rendering primary closure unfeasible. Several fasciocutaneous and myocutaneous flaps of the abdomen and thigh can be used for the reconstruction of the inguinal region, transferring tissue to the affected area, and enabling tensionless closure.

**Conclusions:**

The reconstruction of defects in the inguinal region with the aid of flaps allows for faster postoperative recovery and reduces the risk of complications. Thus, the patient will be able to undergo potential necessary adjuvant treatments sooner.

## INTRODUCTION

Penile cancer is a rare tumor with a higher incidence in developing countries (1–7). Brazil has one of the highest incidence rates of this neoplasia worldwide. The tumor represents 2% of all types of cancer affecting the male population, with a geographical predominance in the North and Northeast regions of the country (1, 8, 9). This type of cancer is more frequent in the male population over 50 years of age, although it can affect younger men as well (9-15). Squamous carcinoma represents 95% of the cases and its dissemination occurs through the lymphatic system, with initial involvement of the inguinal lymph nodes and later affecting the pelvic lymph nodes (1, 3, 10–18). Hematogenic dissemination occurs in less than 10% of cases (1, 4–6).

The pathophysiological factors are still not completely understood, however, phimosis, low socioeconomic status, and low personal hygiene are relevant risk factors for the development of the disease (7, 10, 11, 15, 19, 20). The Human Papilloma Virus (HPV) is involved in 30-50% of all cases (6, 20, 21). In an epidemiological study, Favorito and colleagues (8) found that more than 90% of the cases diagnosed in the Brazilian population originated from the public health system. The low level of education and the difficulty in accessing healthcare hinder early diagnosis and delay treatment start (13). About a third of penile cancers are diagnosed at the stage of locally advanced disease (10, 18, 22). As a consequence, tumors with large lymph node involvement become more frequent.

The size of the tumor and the degree of tumor differentiation are the main predictors of lymph node metastasis (1, 5, 13, 17, 22, 23). About 10-25% of patients with negative physical examination present micrometastases in the histopathological analysis of inguinal lymphadenectomy (1, 12, 15, 17, 24). The presence of lymph node metastasis is the main prognostic factor for patient survival (1, 4, 5, 11, 13, 18, 25, 26).

Radical inguinal lymphadenectomy, encompassing the superficial and deep lymph node chains, is indicated as treatment for patients with diagnosed lymph node metastasis and prophylactically for patients with risk factor for lymph node metastasis (1, 3, 6, 15, 17, 24, 27). It is a procedure that presents itself with a high risk of complications such as skin necrosis, seroma, scrotal and lower limb edema, infection, lymphorrhea, lymphocele and thrombophlebitis (3, 10, 11, 25, 28, 29). The incidence of major complications can reach 40-55% (18, 24, 30).

The aim of this study is to review the surgical alternatives for inguinal reconstruction using flaps after inguinal lymphadenectomy in metastatic penile cancer.

## MATERIALS AND METHODS

**Figure 1:**
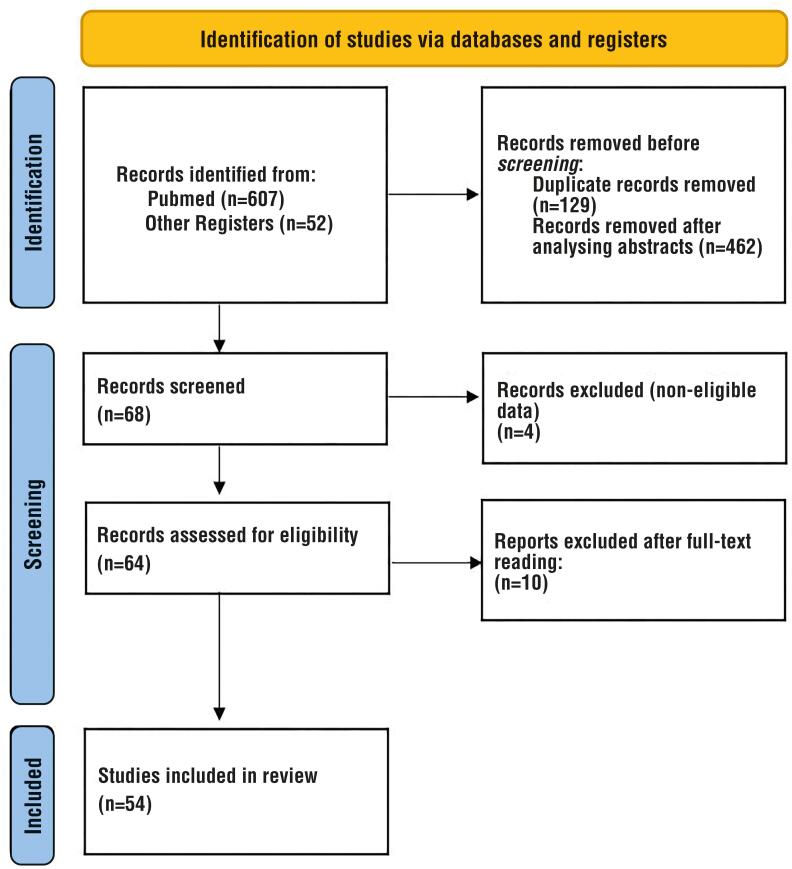
PRISMA flow diagram.

We carried out an extensive literature review according to the PRISMA guidelines using the Pubmed database (Figure-1). We limited the articles selected to publications in English, including reviews and systematic reviews, published between 2010 and 2020. We analyzed papers published in the past 60 years in the databases of Pubmed, Embase and Scielo, found by using the key expressions “advanced penile cancer”, “groin reconstruction”, and “inguinal reconstruction”. We also retrieved and reviewed the clinical guidelines of the websites of the National Cancer Institute (INCA-Brazil), National Cancer Institute (NCI-USA), and the European Association of Urology (UAE). Furthermore, we analyzed the bibliographic references in the selected articles to include new articles in our research.

## RESULTS

With the outlined strategy and using the search terms individually or in combination, we identified 607 articles in the initial research. Of these publications, we excluded 129 studies due to duplicate reporting. A total of 462 articles were analyzed and excluded after evaluating the titles and abstracts. We reviewed the remaining 16 articles. Of these remaining ones, 4 studies were excluded due to the lack of eligible data. Together with the articles selected from the bibliographic references of the articles analyzed, a total of 54 reports were finally selected for this review (Figure-1).

### Penile Cancer

Penile cancer is a disease that carries a very strong social stigma, which contributes to delayed diagnosis and favors the development of locally advanced disease (15, 18, 20, 31). The main justifications for patients to delay seeking medical help are the lack of knowledge about the disease, the fear of severe illness, and the embarrassment of it being an injury to a sexual organ (31).

Currently, new strategies are discussed to reduce lymphadenectomy morbidity, improve survival, and reduce the risk of disease recurrence (7, 19, 26). The use of positron emission tomography - Computed Tomography (PET-CT) in the evaluation of patients with suspected lymphatic involvement has a sensitivity and specificity of 96% and 100%, respectively (16, 17). An alternative is the evaluation of the sentinel lymph node, which helps in the diagnosis and allows for better selection of patients that are candidates for lymphadenectomy (11, 16, 17).

A less frequent presentation of the patient with metastatic penile cancer is cutaneous involvement in the region of lymph node metastasis, which can progress to local ulceration (10, 22). In these cases, the goal of treatment is local control of the disease to prevent complications such as vascular erosion and exsanguination (10, 22, 28). Patients should be evaluated with regards to the extent of the disease, symptoms, and life expectancy before the operative decision. In some cases, neoadjuvant chemotherapy is indicated in an attempt to regress the tumor (7, 20). The metastasis resection should encompass 3-4 centimeters (cm) of disease-free skin, resulting in complex defects to be reconstructed (10, 22).

### Myocutaneous and fasciocutaneous flaps

Defects in the inguinal region resulting from the treatment of metastatic penile cancer are challenging for the surgeon and cause great morbidity (1, 32, 33). Adequate coverage of noble structures, such as femoral vessels, and synthetic materials, such as vascular prostheses, is necessary (15, 34). Because it is a difficult region to keep clean and dry, and subject to tension due to walking and mobility of the lower limb, primary closure is generally not an option (35–37). It is essential to transfer a soft-tissue flap to close the defect without tension, fill in the dead space, and include well-vascularized tissue, allowing for better healing and a reduction of local complications such as dehiscence and infection (10, 32, 35, 38, 39). Scar delay and chronic wounds are common as a consequence of the high incidence of bacterial contamination and local pressure in the inguinal region, favoring ischemia and necrosis (33, 36, 37, 40, 41). Another important factor that interferes with healing is the cachexia often present in patients with advanced tumors (36).

Multiple fasciocutaneous and myocutaneous flaps are used for reconstructing wounds resulting from large penile or lymph node resections. Several flaps of the abdomen and thigh can be transferred to close the defect (32). The most commonly used are the tensor fascia lata myocutaneous flap (TFL), the anterolateral thigh flap and the vertical rectus abdominis myocutaneous flap (VRAM) (28). Flaps from the rectus femoris, gracilis, and sartorius muscles are viable options in cases of defects with less skin loss.

### Donor-site: Thigh

**Figure 2:**
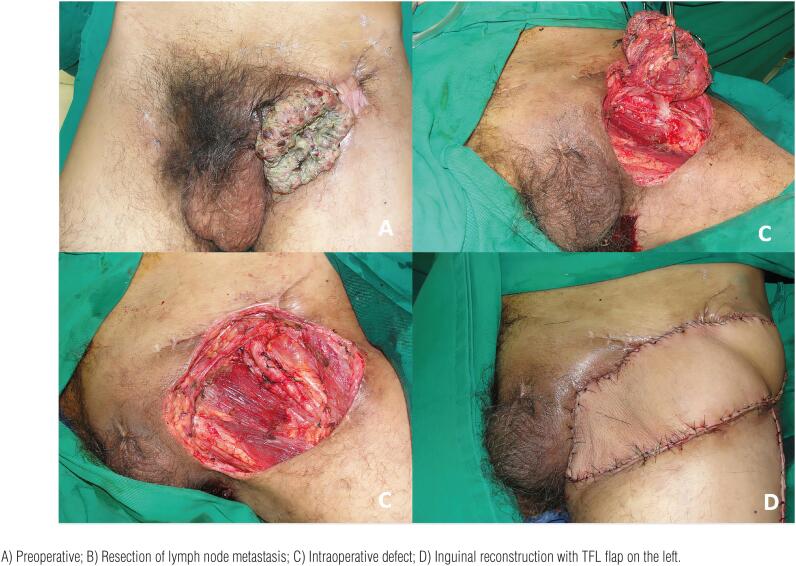
Reconstruction of the inguinal region with a tensor fascia lata myocutaneous flap in a 50-year old patient. The patient underwent resection of the left lymph node metastasis.

**Figure 3 f3:**
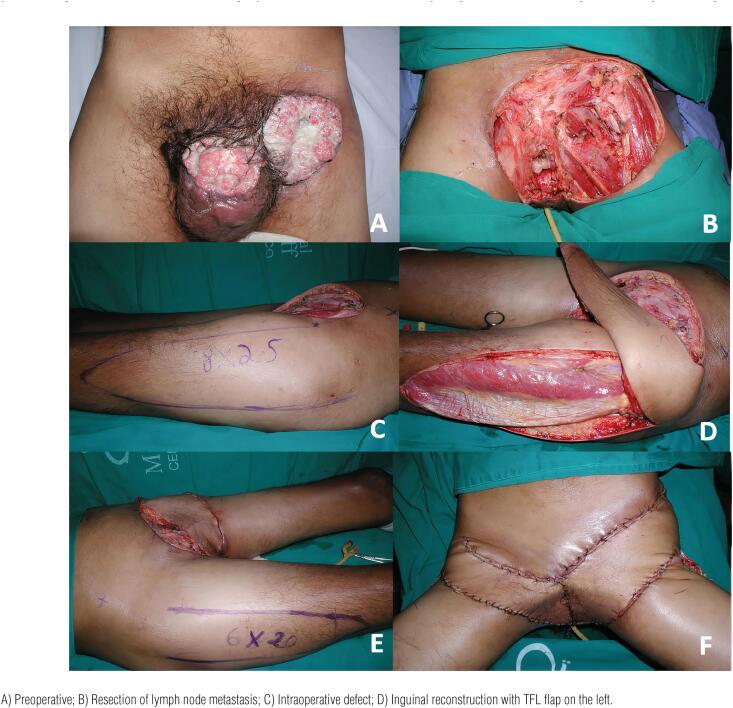
Reconstruction with a bilateral tensor fascia lata myocutaneous flap in a 47-year-old patient. The patient underwent penectomy and resection of the left lymph node metastasis causing major defect in the inguinal and genital regions.

**Figure 4 f4:**
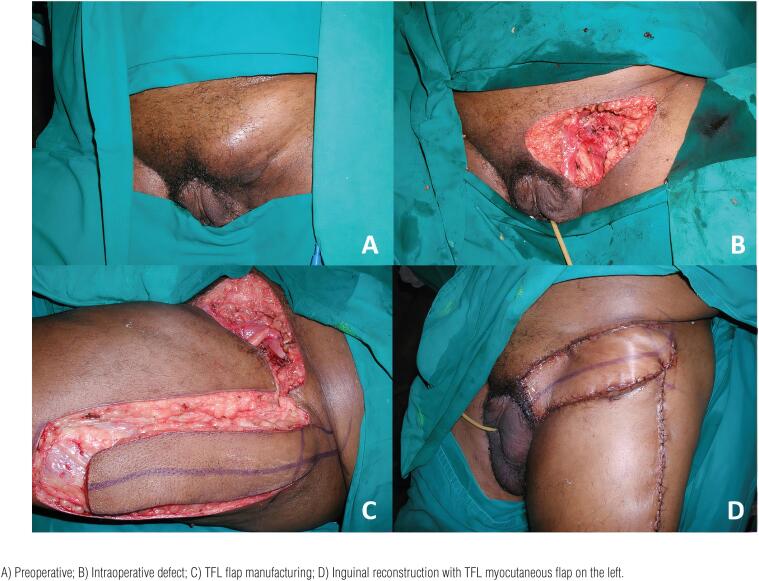
Reconstruction of the inguinal region with a tensor fascia lata myocutaneous flap in a 69-year-old patient. The patient underwent resection of the left lymph node metastasis.

**Figure 5:**
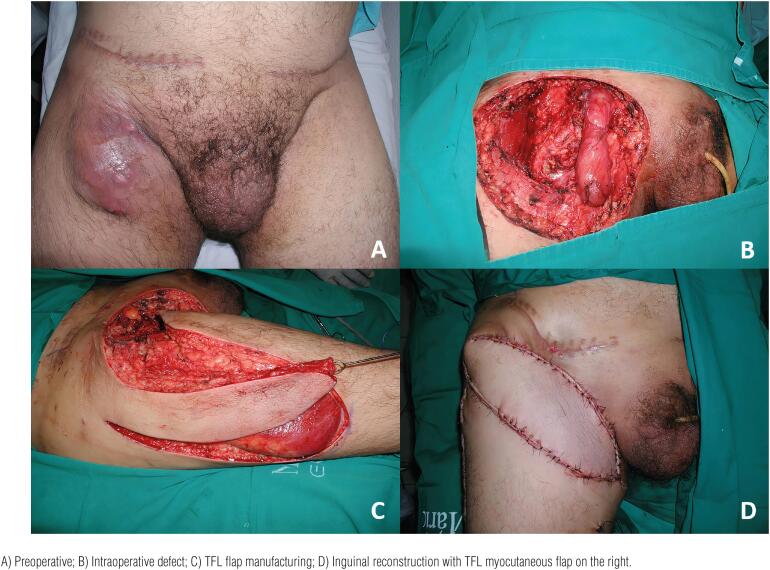
Reconstruction of the inguinal region with a tensor fascia lata myocutaneous flap in a 40-year-old patient. The patient underwent resection of a lymph node metastasis on the right.

The TFL myocutaneous flap is a versatile flap and an excellent option for the reconstruction of defects in the inguinal and lower abdominal regions through transferring a skin paddle associated with a strong fascia (10, 28, 33, 38, 42). It has a vascular pedicle with constant anatomy: the ascending branch of the lateral femoral circumflex artery. It is an easy-to-make flap with a skin paddle of an adequate size for most defects, excellent arc of rotation, and low morbidity for the donor site. The flap can be designed up to 10-12cm wide, allowing primary closure of the donor area (28, 38). For larger defects it can be extended with skin grafting in the donor area or performed in combination with other myocutaneous flaps. The flap can reach up to 15x40cm (42). The lower limit of the TFL skin island should be 8-10cm from the knee since longer flaps are unreliable. The literature shows that the incidence of partial flap necrosis can vary from 10-50% (28, 38). (Figures 2–5) [Fig f4]

The anterolateral thigh flap can provide good coverage for the inguinal and lower abdominal region (36, 43). It is based on the perforators of the descending branch of the lateral femoral circumflex artery, with the perforators of the flap located halfway between the anterior superior iliac crest and the superolateral edge of the patella, concentrating in a radius of 3-5cm to that reference point (28, 30, 36, 44). The flap can be lifted with several components, including skin, subcutaneous, muscle, nerve, and the fascia of the tensor fascia lata muscle, which is an advantage in cases that involve defects of the lower abdominal wall (28, 43, 44). It can be tunneled to the inguinal region through a tunnel in the subcutaneous or deep to the rectus femoris and sartorius muscles to increase the length of the pedicle (36, 37, 43, 45). It is an excellent option because of its proximity to the donor area and because of the long and constant pedicle that can reach 14-16cm in length (30). It causes low morbidity at the donor site, however some studies report temporary paresis of the lower limb, which usually regresses completely within 6 months (33, 34, 43, 46, 47).

The gracilis muscle flap is an option of flap that can be transferred with or without a skin paddle (39, 47). It can be an alternative in cases where the rectus abdominis musculature is involved and renders the VRAM flap an unviable option (40, 48). The gracilis muscle flap has a main pedicle based on the ascending branch of the medial femoral circumflex artery and segmented secondary pedicles derived from branches of the superficial femoral artery (36, 47). Some studies have shown a high incidence of partial necrosis, reaching up to 38% of cases (30, 39, 40, 49). The flap skin island is drawn along the upper two thirds of the gracilis muscle, the location of the musculocutaneous perforators. The flap dissection should include the adjacent fasciocutaneous perforators of the adductor muscles to increase the viability of the flap (36, 39, 49). As its pedicle is limited in length, it is less often used for inguinal reconstruction (37). Its restricted volume and the potential complications at the donor site make its use less common.

The rectus femoris myocutaneous flap offers a favorable arc of rotation for transposition into the inguinal region (39, 50, 51). It is easily elevated after an anterior medial incision in the distal two thirds of the thigh with disinsertion of its distal patellar portion. The flap is elevated in a proximal direction until the identification of its pedicle, the descending branch of the lateral femoral circumflex artery (51). The flap is transferred to the inguinal region through a subcutaneous tunnel connecting the donor area to the defect (39, 50). Although the rectus femoris muscle is narrow, with only 6cm wide, it allows for the transfer of a cutaneous segment of up to 12-15cm (51). The donor area is closed primarily or through partial skin grafting. Many authors report to be afraid to use this flap due to the potential loss of strength in the knee extension (39, 50, 51). This reduction in quadriceps strength can reach 24-28% (39).

The sartorius muscle flap was originally described to cover femoral vessels and obliterate the dead space after inguinal lymphadenectomy (27, 30). This flap is an option for reconstruction of inguinal defects when there is no need for skin island transfer (39). Its proximity to the area to be reconstructed is an advantage, however, its transposition is limited due to the particularity of its pedicle (39, 52). The flap has segmental vascular pedicles composed of six to seven branches of the superficial femoral artery, a characteristic that restricts the flap size and its rotation arc (30, 39, 50, 52).

**Figure 6:**
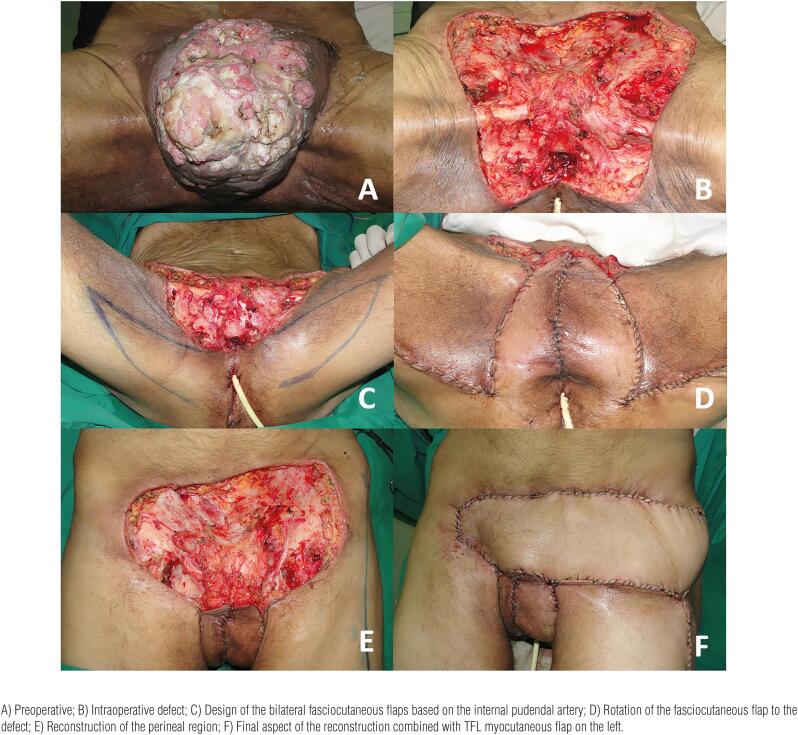
Reconstruction with bilateral fasciocutaneous flap based on the internal pudendal artery associated with a tensor fascia lata myocutaneous flap in a 63-year-old patient. The patient was submitted to penectomy and resection of bilateral lymph node metastasis that caused major defect in the inguinal and perineal regions.

The fasciocutaneous flap of the medial aspect of the thigh has its vascular pedicle located in the cutaneous projection of the ischial tuberosity. When the internal pudendal artery emerges under the ischial tuberosity, it sends cutaneous branches to the inner side of the thigh and forms a rich anastomotic network, increasing the flap's reliability (48, 53). The reference point for the flap design is the cutaneous projection of the ischial tuberosity. The longest flap axis can extend to the triangular thigh fossa, while the flap width will depend on the region's bigital clamping maneuver to allow primary closure of the donor area without tension. The flap can reach 15x8cm, including skin, subcutaneous tissue, and the epimysium of the adductor musculature. In most cases, the flap is elevated bilaterally (47, 48). (Figure-6)

### Donor-site: Lower abdomen

Historically, the VRAM flap is one of the main alternatives for the reconstruction of pelvic, inguinal, and perineal defects (32, 36, 43, 47). Its main advantages are a reliable vascularization, the transfer of a large skin paddle, and its muscle volume for the closure of large dead spaces (29, 32, 35, 36, 47, 54). The flap pedicle is based on the deep inferior epigastric artery, the dominant artery in the abdominal wall. The flap can be ipsilateral or contralateral, depending on the surgeon's preference or limitation of previous scars or ligation of the flap's nourishing vessels (32, 35, 36, 43). In the traditional VRAM flap, the skin island is designed centered on the rectus abdominis muscle that will be lifted (28, 32, 36). In cases of large defects, the flap can be modified and transferred as an extended flap, drawn obliquely towards the midaxillary line. The extended VRAM flap can take up to 40 x 9cm in size (28, 35, 37).

The dissection of the VRAM flap is done carefully to preserve the largest number of medial and lateral perforators. The rectus abdominis muscle is incised superiorly and raised in connection to a narrow band of the anterior sheath of the musculature. Preservation of one centimeter lateral and medial of the anterior sheath reduces the incidence of bulging of the abdominal wall and hernia (35). The anterior fascia can be closed primarily or with the aid of a mesh (28, 35). In the literature, the incidence of complications at the donor site ranges from 10-40% (32, 37, 43).

The transfer of perforating flaps to the inguinal region is an advantageous alternative as it reduces the morbidity of the donor site when harvesting the flap without harming the adjacent musculature and its main vessels (39, 45). The perforating flap of the deep lower epigastric artery reduces the morbidity of the abdominal wall, but its skin island is considerably smaller than the one of the VRAM flap (34, 43).

### Free Flaps

The use of microsurgical flaps is also a possibility for these reconstructions, however, the use of pedicled flaps reduces the operative time, usually don't require a change in the patient´s position, and avoid the dissection of vessels that may suffer damage with radiotherapy (28, 35). The microsurgical technique should be reserved for cases where flaps of the abdomen or thigh cannot be used given insufficient pedicle length or excessive pedicle tension (37).

## CONCLUSIONS

A successful reconstruction in metastatic penile cancer depends on detailed surgical planning involving the Urology and Plastic Surgery teams. The reconstruction of defects of the inguinal region with the aid of flaps contributes to faster postoperative recovery, allows for early ambulation and reduces the risk of complications (28). The use of myocutaneous flaps additionally has the benefit of minimize local morbidity in cases where radiotherapy is associated with treatment, as it reduces the risk of infections and delayed healing (32, 37, 38, 47). The shorter the recovery, the faster the patient will be able to undergo adjuvant treatments if necessary.

## ABREVIATIONS

cm: = centimeter
INCA: = Instituto Nacional do Câncer
NCH: = National Cancer Institute
EAU: = European Association of Urology
PET-CT: = Positron Emission Tomography - Computed Tomography
TFL: = tensor fascia lata myocutaneous flap
VRAM: = vertical rectus abdominis myocutaneous flap
